# Distribution and origins of *Mycobacterium tuberculosis* L4 in Southeast Asia

**DOI:** 10.1099/mgen.0.000955

**Published:** 2023-02-02

**Authors:** Philip M. Ashton, Jaeyoon Cha, Catherine Anscombe, Nguyen T. T. Thuong, Guy E. Thwaites, Timothy M. Walker

**Affiliations:** ^1^​ Oxford University Clinical Research Unit, Ho Chi Minh City, Vietnam; ^2^​ Centre for Tropical Medicine and Global Health, Nuffield Department of Medicine, University of Oxford, Oxford, UK; ^3^​ Institute of Infection, Veterinary and Ecological Sciences, University of Liverpool, Liverpool, UK; ^4^​ Department of Molecular Biology, Princeton University, Princeton, New Jersey, USA

**Keywords:** tuberculosis, genomics, phylogeography

## Abstract

Molecular and genomic studies have revealed that *Mycobacterium tuberculosis* Lineage 4 (L4, Euro-American lineage) emerged in Europe before becoming distributed around the globe by trade routes, colonial migration and other historical connections. Although L4 accounts for tens or hundreds of thousands of tuberculosis (TB) cases in multiple Southeast Asian countries, phylogeographical studies have either focused on a single country or just included Southeast Asia as part of a global analysis. Therefore, we interrogated public genomic data to investigate the historical patterns underlying the distribution of L4 in Southeast Asia and surrounding countries. We downloaded 6037 genomes associated with 29 published studies, focusing on global analyses of L4 and Asian studies of *M. tuberculosis *. We identified 2256 L4 genomes including 968 from Asia. We show that 81 % of L4 in Thailand, 51 % of L4 in Vietnam and 9 % of L4 in Indonesia belong to sub-lineages of L4 that are rarely seen outside East and Southeast Asia (L4.2.2, L4.4.2 and L4.5). These sub-lineages have spread between East and Southeast Asian countries, with no recent European ancestor. Although there is considerable uncertainty about the exact direction and order of intra-Asian *M. tuberculosis* dispersal, due to differing sampling frames between countries, our analysis suggests that China may be the intermediate location between Europe and Southeast Asia for two of the three predominantly East and Southeast Asian L4 sub-lineages (L4.2.2 and L4.5). This new perspective on L4 in Southeast Asia raises the possibility of investigating host population-specific evolution and highlights the need for more structured sampling from Southeast Asian countries to provide more certainty of the historical and current routes of dispersal.

## Data Summary

All the genome sequence data used in this project can be publicly accessed from NCBI/ENA/DDBJ. The accession numbers are available in Table S1 (available with the online version of this article). Furthermore, intermediate files can be accessed from FigShare. The input and output files for TreeBreaker can be accessed at https://doi.org/10.6084/m9.figshare.21378312, the files for replicating the iTOL trees can be accessed at https://doi.org/10.6084/m9.figshare.21378330.v1, and the files for the TreeTime analysis can be accessed at https://doi.org/10.6084/m9.figshare.21401307.v1. Code to parse the TreeBreaker output file can be accessed at https://gist.github.com/flashton2003/50d645a60219c0e381874a1dd4355646.

**Impact Statement**

This article combines data from 29 different publications to improve our understanding of the dispersal of *Mycobacterium tuberculosis* Lineage 4 (L4), one of the most globally important lineages of *M. tuberculosis *. We found that L4 has been dispersed between Asian countries for hundreds of years, which extends our idea of L4 as the ‘Euro-American’ lineage. This work provides a platform for further research into the potential host adaptation of Asian sub-lineages of L4 that have been circulating in East/Southeast Asian populations for hundreds of years.

## Introduction

*Mycobacterium tuberculosis* caused 5.8 million reported cases of tuberculosis (TB) and 1.5 million reported deaths in 2020 [1]. The significant disruption to TB services from the COVID-19 pandemic mean the true numbers are likely to be much higher [1, 2]. Molecular and genomic studies have revealed that there are at least eight lineages of *M. tuberculosis *, which display variable degrees of phylogeographical signal [3–5].

*M. tuberculosis* Lineage 4 (L4) is globally distributed [6], and is thought to have originated in Europe [7–9]. Colonialism and long-distance trade have been proposed to be important for the spread of L4 to the Americas, Africa, Asia and Oceania [9, 10]. While Lineage 2 (Beijing lineage) is the most prevalent lineage in most countries in East and Southeast Asia, the high TB burden in these countries means that the absolute number of TB cases caused by L4 isolates is nevertheless large. Much of our understanding of the molecular epidemiology of LB in the region is derived from spoligotyping. This showed that Indonesia had the highest proportion of L4 in Southeast Asia, with publications focusing on different parts of the country reporting 28–47 % L4 [6, 11–14]. Analysis of the spoligotype of 16 621 isolates from all 32 provinces of China found that L4 accounted for around 17 % of *M. tuberculosis* [15]. A lower prevalence of L4 is seen across Vietnam (6.4–12.2 % [6, 16, 17]), Myanmar (8 % [18]), Thailand (10 % [6]) and Malaysia (9.6–13.5 % [6, 19]). Reports from Cambodia and the Philippines show lower rates still (0–1 % [20, 21] and 1 % [22] respectively).

Studies making use of whole genome sequencing (WGS) have recently improved our understanding of L4 in East and Southeast Asia. A well-structured sampling of 279 L4 genomes from China revealed that 97 % of L4 in China belongs to one of three L4 sub-lineages (L4.2.2, L4.4.2 and L4.5), which were introduced from Europe between the 11^th^ and 13^th^ centuries, probably mediated by the intense trade connections during this historical period, exemplified by the Maritime Silk Road [15]. From Southeast Asia, only Vietnam, Indonesia, Thailand and the Philippines have more than 10 published L4 genomes [23–26]. The L4 genomes from Vietnam came from a study of the genomic epidemiology of *M. tuberculosis* in Ho Chi Minh City, and a study of drug-resistant *M. tuberculosis* in Hanoi [25, 27]. The currently available L4 genomes from Indonesia came from the city of Bandung on the island of Java as part of a study examining differences between *M. tuberculosis* causing pulmonary TB and TB meningitis [24]. L4 genomes from Thailand came from studies comparing pulmonary TB and TB meningitis and a cohort study [23, 28]. The analyses from Indonesia and Thailand did not include any international genomes for context, so their phylogenetic relationship to the broader diversity of L4 is unknown [23, 24].

However, our understanding of L4 in East and Southeast Asia remains piecemeal as no studies to date have combined all published datasets for analysis. Therefore, to investigate the historical patterns underlying the present distribution of L4 in Southeast Asia, we carried out a combined analysis of published *M. tuberculosis* L4 genomes from East and Southeast Asia, along with contextual genomes from global data sets.

## Methods

### Data download

We carried out a literature review to identify papers that reported either *M. tuberculosis* genomes from Southeast Asia or globally representative *M. tuberculosis* L4 genomes. We used this search strategy to identify as many Southeast Asian L4 genomes as possible (by including all studies from the region), while also including representatives of global diversity.

### Data processing

Downloaded data were processed with bbduk v38,96 [29] in order to remove adapters and low-quality sequencing regions: ‘bbduk.sh ref=adapters.fa in=!{forward} in2=!{reverse} out=!{pair_id}_bbduk_1.fastq.gz out2=!{pair_id}_bbduk_2.fastq.gz ktrim=r k=23 mink=11 hdist=1 tbo tpe qtrim=r trimq=20 minlength=50’. Trimmed fastqs files were then analysed with tb-profiler [30] in order to identify the lineage of each readset. Trimmed fastqs files were also mapped against the H37Rv reference genome (NCBI accession NC000962.3) using bwa mem v0.7.17-r1198-dirty [31]. SNPs were called with GATK v3.8-1-0-gf15c1c3ef in unified genotyper mode [32]. Positions where the majority allele accounted for <90 % of reads mapped at that position, which had a genotype quality of <30, depth <5× or mapping quality <30 were recorded as Ns in further analyses. A consensus genome was generated for each genome. These steps were carried out using the PHEnix pipeline [33].

### Phylogenetics and phylogeography

After consensus genomes were combined, we used snp-sites v2.5.1 to extract the variant positions, and then generated a neighbour-joining tree of all 6037 samples with IQ-TREE v2.1.4-beta [34]. The tb-profiler results were combined with the neighbour-joining tree and the L4 genomes identified. A maximum-likelihood phylogenetic tree of the L4 genomes was then derived using IQ-TREE with built-in model selection, and the inclusion of the number of invariant sites, as identified using snp-sites. TreeBreaker v1.1 [35] was used to identify internal nodes of the tree where there was a change in the distribution of phenotypes of interest at the tips that descended from that internal node. The TreeBreaker command line used was ‘treeBreaker -x 5000000 -y 5000000 -z 10 000 input.tree phenotype.txt output_prefix’. The phenotype of interest was the geographical location. To enable easy interpretation, separate TreeBreaker runs were carried out for Vietnam, Indonesia, China and Thailand, and all the preceding countries combined into a single category (i.e. a single ‘phenotype’ of belonging to either Vietnam, Indonesia, China or Thailand). TreeBreaker outputs a text file that, on the last line of the file, has a newick format phylogenetic tree with the results annotated onto the internal nodes. This newick tree was extracted from the text file and saved as a tree file. It was then converted to a nexus format tree using FigTree (ensuring to include annotations) for reading into dendropy v4.5.2 [36]. The nexus format tree was then parsed using a script (https://gist.github.com/flashton2003/50d645a60219c0e381874a1dd4355646) to produce sub-trees and summary information for nodes above the 0.5 posterior probability threshold. Example input and output files for TreeBreaker analysis can be accessed from https://doi.org/10.6084/m9.figshare.21378312. As TreeBreaker produces results annotated onto the nodes of the input phylogenetic tree, and we used the same input tree for all analyses, we could combine the results from these different runs based on the identifiers of the internal nodes. As we were using TreeBreaker as a screening tool, to identify nodes for further analysis using SIMMAP, we filtered for nodes with a posterior probability threshold of 0.5 and at least five descendent leaves. All SIMMAP analysis [37] was carried out using the make.simmap function from PhyTools [38] in the R statistical language [39] using RStudio [40]. The fit of each model type (all rates different, symmetrical and equal rates) was assessed using the fitMk function, and the model with the best fit was used for the SIMMAP analysis. We ran 1000 simulations within SIMMAP. Nodes that were identified as being associated with changes by TreeBreaker were targeted for investigation in the output of SIMMAP. Trees (newick format) were visualized with iTOL [41], and graphs drawn with ggplot2 [42]. The files for replicating the iTOL trees can be downloaded from https://doi.org/10.6084/m9.figshare.21378330.v1. Phylotemporal analysis was carried out using TreeTime v0.9.0 [43] with a substitution rate and standard deviation of 0.000000061643 and 0.0000000385, respectively. These values were obtained from the estimates of the ‘BEAST constant population size, uniform prior on clock rate’ analysis of Menardo *et al*. [44]. The command line used was ‘treetime –clock-rate 0.000000061643 –tree input.tree –dates input_dates.csv –outdir my_analysis –sequence-length 4411532 –confidence –clock-std-dev 0.0000000385’. Input data for TreeTime analysis can be found at https://doi.org/10.6084/m9.figshare.21401307.v1.

### Sub-sampling

To investigate whether phylogeographical results identified in the full dataset were robust to differences in sampling, we sub-sampled 20 genomes from each of China, Thailand, Indonesia and Vietnam, and all the European genomes available for each sub-clade, and carried out phylogeographical analysis using the TreeTime mugration command [43], and the location of the most recent common ancestor (MRCA) of the Asian sub-clade was extracted. This sub-sampling and phylogeographical analysis was repeated 1000 times.

## Results

We identified 6037 read-sets associated with 29 publications on *M. tuberculosis* genomics [8, 9, 15, 23–28, 45–65] and downloaded them from the European Nucleotide Archive (ENA). We identified 2257 L4 read-sets that were not mixed (i.e. where only a single L4 sub-lineage was identified) and that had associated geographical information (Tables 1 and S1). Of the 2257 read-sets included in this analysis, 968 (43 %) were from Asia, 581 (26 %) were from Europe, 501 (22 %) were from South America, 144 (6 %) were from Africa, 58 (3 %) were from North America and five (0.2 %) were from Oceania (Tables S1 and S2). We identified 308 read-sets as belonging to the L4.3.3 sub-lineage, 268 as L4.5, 255 as L4.1.2.1, 237 as L4.8, 156 as L4.3.4.2, 140 as L4.4.2, 110 as L4.2.2 and 784 belonging to other L4 sub-lineages (Tables S1 and S2). As a note on terminology, we use the term ‘sub-lineage’ to refer to a group of L4 *M. tuberculosis* genomes with a shared Coll *et al*. designation, e.g. L4.4.2 [66], and ‘sub-clade’ to refer to a monophyletic part of a sub-lineage. A sub-clade may have a different geographical distribution than the overall sub-lineage. There were three East/Southeast Asian sub-lineages identified – L4.2.2, L4.4.2 and L4.5, that were 79, 99 and 97 % from East/Southeast Asia (Tables S1 and S2). East/Southeast Asian sub-lineages are defined as ≥75 % of the sub-lineage in our collection being from East or Southeast Asia, and where there were more than 50 genomes from that sub-lineage in our analysis.

**Table 1.** The number of genomes from each country included in this study, and the studies they were first reported in

Country	No.	Studies
UK	337	Casali *et al*., 2012, *Genome Res* [72]; Casali *et al*., 2014, *Nat Genet* [73] ; Walker *et al*., 2013, *LID* [45]; Walker *et al*., 2014, *LID* [46]
Vietnam	275	Comas *et al*., 2013, *Nat Genet* [47]*;* Holt *et al*., 2018, *Nat Genet* [25]; Maeda *et al*., 2019, *Infection, Genetics and Evolution,* [27] *Stucki et al., 2016, Nat Genet* [8]
Brazil	263	Brynildsrud *et al*., 2018 *Sci Adv*, [9]
Peru	230	Grandjean *et al*., 2017, *PLoS ONE* [74]
Indonesia	194	Ruesen *et al*., 2018, *BMC Genomics* [24] *;* Stucki *et al*., 2016, *Nat Genet* [8]
Thailand	189	Ajawatanawong *et al*., 2019, *Sci. Rep.* [28] ; Bryant *et al*., 2013, *Lancet Resp. Med*. [48]; Faksri *et al*., 2018, *Sci. Rep*. [23]; *Stucki et al., 2016, Nat Genet* [8]
China	181	*Comas et al., 2013, Nat Genet* [47]; Liu *et al*., 2018, *Nat. Eco. Evo* [15]; Stucki *et al*., 2016, *Nat Genet* [8]; Zhang *et al*., 2013, *Nat Genet* [49],
Netherlands	112	Bryant *et al*., 2013, *BMC Infectious Diseases* [62]
Russia	73	*Casali et al., 2012, Genome Res* [72]; *Casali et al., 2014, Nat Genet* [73]
Congo	57	Malm *et al*, 2017, *EID* [75]
Malawi	45	Guerra-Assunção *et al*., 2015, *eLife* [65]
Portugal	44	Perdigão, 2014 *BMC Genomics* [63]
Canada		Pepperell *et al*., 2011, *PNAS* [68]; Brynildsrud *et al*., 2018, *Sci Adv* [9]
India	36	Advani *et al*., 2019, *Frontiers in Microbiology* [50]; Chatterjee *et al*., 2017, *Tuberculosis* [51]; Manson *et al*., 2017, *CID* [52]; Shanmugam *et al*., 2019, *MRA* [53]; *Stucki et al., 2016, Nat Genet* [8]
Philippines	31	Phelan *et al*., 2019, *Scientific Reports* [26]; *Stucki et al., 2016, Nat Genet* [8]
Uganda	28	Clark *et al*., 2013, *PLoS ONE* [76]; Comas, 2013, *Nat Genet* [47]
Other	120	

**Fig. 1.**
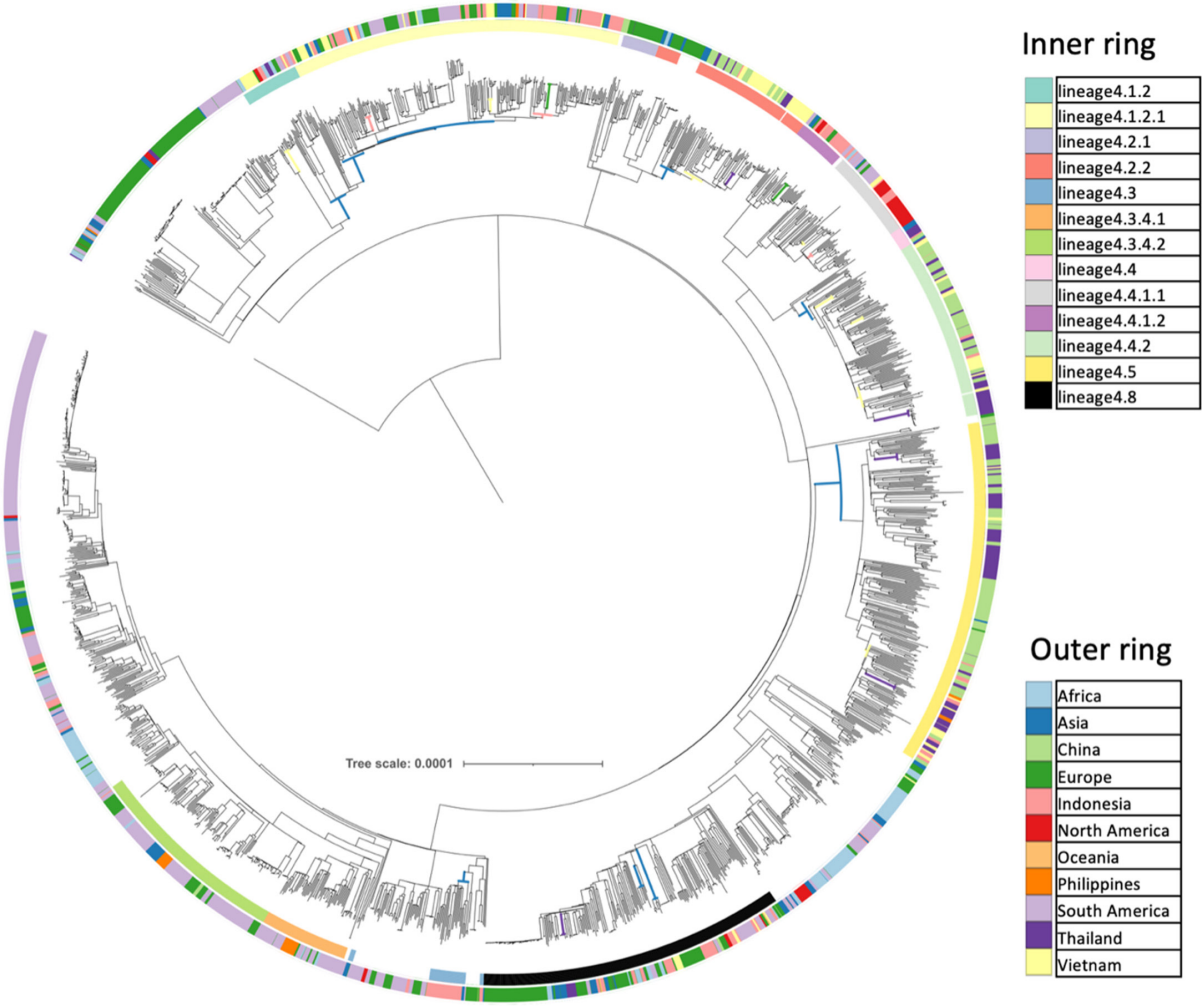
A maximum-likelihood phylogenetic tree of 2258 *M. tuberculosis* L4 genomes (the 2257 read-sets identified and the H37Rv reference genome). The inner annotation ring indicates the Coll et al., 2014 sub-lineage, and the outer ring indicates the geographical area of origin. For clarity, we only highlighted sub-lineages that are discussed further in this paper. Internal branches are coloured when both TreeBreaker and SIMMAP analysis identified a change between geographical areas of interest on that branch; the colour of the branch represents the country or region that SIMMAP identified as the location of the MRCA node.

We constructed a maximum-likelihood phylogenetic tree of 2258 L4 genomes (2257 read-sets and the H37Rv reference genome, Fig. 1) and used it as the input for TreeBreaker [35] analysis. Four TreeBreaker analyses were carried out, all with a binary phenotype, for Vietnam (i.e. was the isolate from Vietnam or not), Indonesia, Thailand, and a combined analysis where Vietnam, Indonesia, Thailand and China were grouped together into a single phenotype. This identified 10 sub-clades for Indonesia (Fig. S4), 10 sub-clades for Thailand (Fig. S5), 15 sub-clades for Vietnam (Fig. S6) and 19 sub-clades for the combined Vietnam, Indonesia, Thailand and China analysis (Fig. S7). When the sub-clades were de-duplicated (based on the internal node from which it descended), there were a total of 40 sub-clades with changes in the proportion of tips coming from our countries of interest (see Methods for details, Table S3). TreeBreaker identified sub-clades belonging to L4.5 (*n*=7), L4.1.2.1 (*n*=7), L4.4.2 (*n*=6), L4.8 (*n*=6), L4.2.2 (*n*=3), L4.4.1.1 (*n*=2), L4.4.1.2 (*n*=2), L4.1.2 (*n*=2), L4.3 (*n*=1), L4.2.1 (*n*=1), L4.4 (*n*=1), L4.3.4.1 (*n*=1) and L4.3.4.2 (*n*=1). We used SIMMAP to infer the geographical location of ancestral nodes in the phylogeny. Internal branches identified by both TreeBreaker and SIMMAP as representing movements into or out of our geographical areas of interest (*n*=27) are highlighted in Fig. 1.

**Fig. 2.**
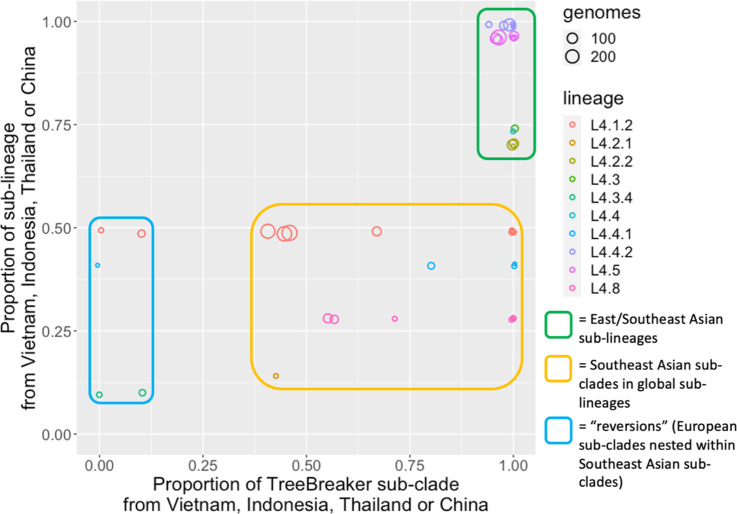
We identified three kinds of L4 sub-clades among those identified by TreeBreaker. The three kinds varied in the proportion of the sub-clade that was present in Vietnam, Indonesia, Thailand or China, and by the proportion of the sub-lineage that was from those countries. Each circle represents a sub-clade descending from an internal branch that TreeBreaker identified as representing a change between two geographical locations. The size of the circle represents the number of genomes in the sub-clade. The circles are not necessarily independent as sub-clades can be nested within higher sub-clades (see Figs S4–S7 for a tree representation of this). The colour of each circle represents the sub-lineage it belongs to. Rectangles with curved corners represent the different kinds of sub-clade identified. The first ‘type’ of sub-clade we identified is Asian sub-clades in Asian sub-lineages, located in the top right of the graph (green rectangle) because a high proportion of this sub-clade is from Asia, and also because the majority of isolates the sub-clades we identified in our TreeBreaker analysis come from Asia. The second type of sub-clade we identified was Asian sub-clades within global lineages (orange rectangle), which represent sub-clades enriched in Asian isolates within sub-lineages that are generally global. Finally, there are ‘reversions’, i.e. non-Asian sub-clades (since the *x*-axis position is close to zero) nested within Asian sub-clades of global sub-lineages. These represent migrations of global L4 sub-clades from Asia back to Europe.

We identified three main kinds of sub-clade in our combined TreeBreaker/SIMMAP analysis: (i) Southeast Asian sub-clades in East/Southeast Asian sub-lineages, (ii) Southeast Asian sub-clades within global lineages and (iii) reversions, which are European sub-clades nested within an East/Southeast Asian sub-clade; these are termed reversions as L4 originated in Europe, and these sub-clades have moved from Europe to Asia, before ‘reverting’ to Europe (Fig. 2).

### East/Southeast Asian sub-lineages

Here we present SIMMAP analysis results for the sub-clades identified by TreeBreaker/SIMMAP in the East/Southeast Asian sub-lineages. In China, 94 % of L4 belonged to the East/Southeast Asian sub-lineages (L4.2.2, L4.4.2, L4.5), in Thailand this was 81%, in Vietnam 51 % and in Indonesia 9 %.

### L4.2.2

**Fig. 3.**
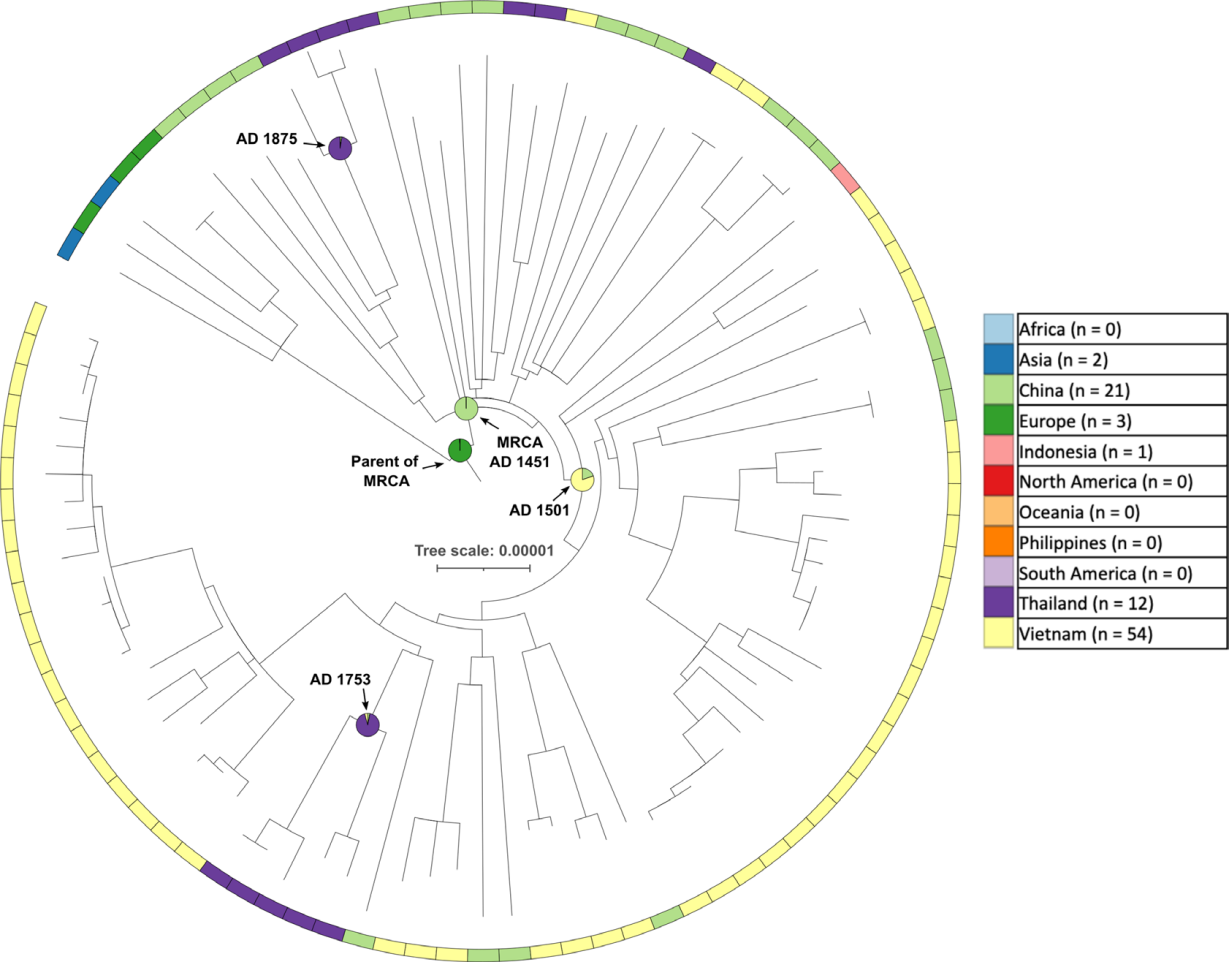
A maximum-likelihood phylogeny of 88 L4.2.2 genomes. SIMMAP analysis indicates that there was migration from Europe to China, and subsequently from China to Vietnam. Pie charts on internal nodes represent the probability of the geographical location of that hypothetical ancestor. Scale bar units are number of substitutions per site.

L4.2.2, one of the East/Southeast Asian sub-lineages, had a sub-clade of 88 genomes identified by TreeBreaker, of which 54 were from Vietnam, 21 were from China, 12 were from Thailand and one was from Indonesia (Fig. 3). SIMMAP analysis showed that the probable geographical location of the MRCA of this sub-clade was in China (99 % probability), and phylogenetic dating analysis gave a date of AD 1451 [95 % confidence interval (CI) 1408–1452]. The parent node of the Chinese MRCA was assigned to Europe (99 % probability). This relationship between the MRCA node and the parent node of the MRCA indicates that, according to the SIMMAP analysis, a migration event from Europe to China occurred at some point along the branch between those two nodes. There was a further sub-clade of 64 genomes, including 51 of the 54 Vietnamese genomes, for which the MRCA was probably in Vietnam (81%) in AD 1501 (95 % CI 1467–1509), while the parent node of the MRCA was placed in China (99%). All of the sub-sampled replicates identified China as the location of the MRCA of L4.2.2.

### L4.4.2

**Fig. 4.**
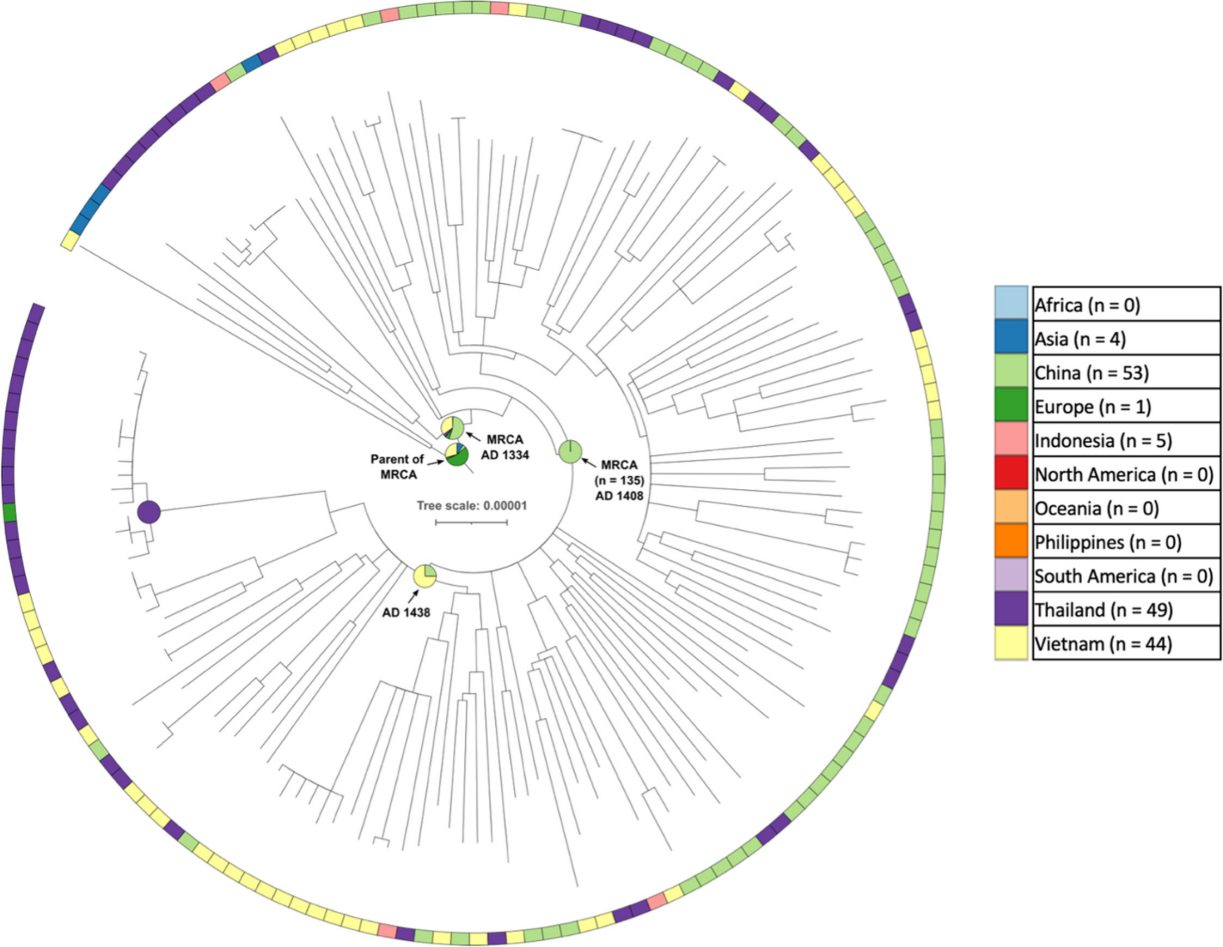
A maximum-likelihood phylogeny of 152 L4.4.2 genomes. SIMMAP assignment of the MRCA of the entire sub-lineage is ambiguous, but there is a sub-clade of 135 genomes (indicated with an arrow) that was assigned to China with high confidence, and then a migration from China to Vietnam, and subsequently from Vietnam to Thailand. Internal node annotations are as per Fig. 3. Scale bar units are number of substitutions per site.

L4.4.2 was another East/Southeast Asian sub-lineage, with 152 genomes in a sub-clade identified by TreeBreaker (including 12 basally branching L4.4 genomes), of which 53 were from China, 49 were from Thailand, 43 from Vietnam, five from Indonesia, one from Malaysia and one from the UK (Fig. 4). SIMMAP produced an ambiguous result for the location of the MRCA of this sub-clade, and of the parent node of the MRCA. The MRCA was in either China (54%) or Vietnam (34%) in AD 1334 (90 % CI 1288–1343), while the parent node of the MRCA was either in Europe (55%) or Vietnam (29%). However, a sub-clade of 135 of the 152 genomes had an MRCA which was assigned to China with a high probability (>99 %) and dated to AD 1408 (90 % CI 1371–1412). Within this there was a clade of 52 genomes for which the MRCA was in either Vietnam (75%) or China (25%) in AD 1438 (90 % CI 1411–1445). In the sub-sampling analysis, 56.8 % of the replicates placed the MRCA in Thailand, 43 % in Vietnam and 0.2 % in China.

### L4.5

**Fig. 5.**
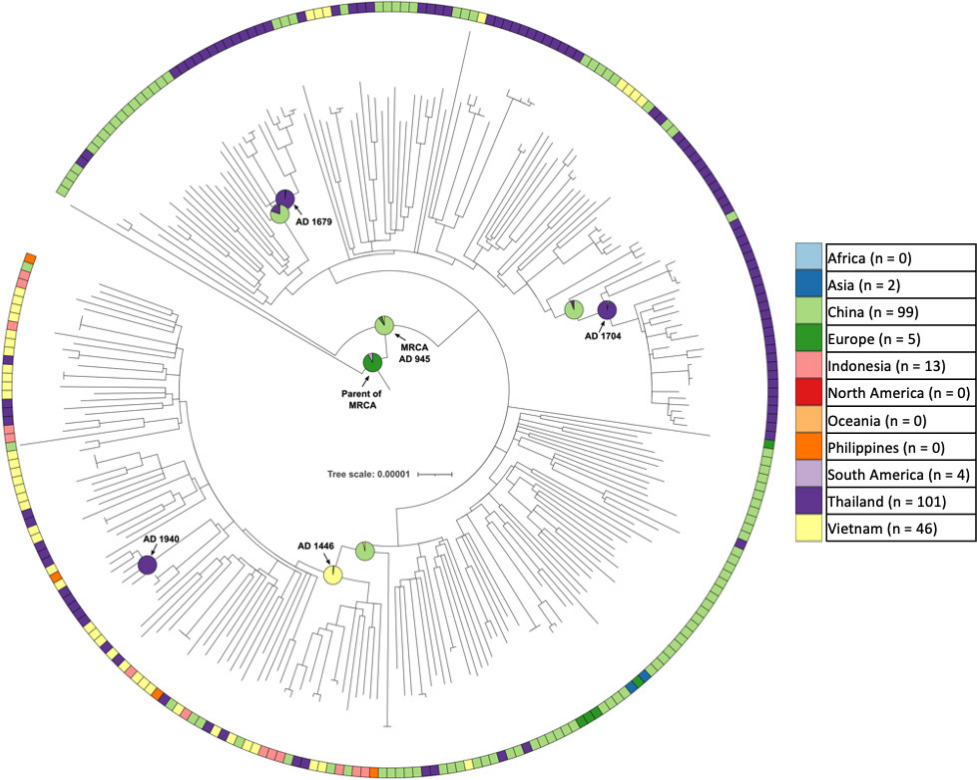
A maximum-likelihood phylogeny of 270 L4.5 genomes. SIMMAP analysis indicates that there was migration from Europe to China. From China there were multiple migrations to Thailand and separately to Vietnam. There were multiple other high-confidence migrations, enumerated in the main text. Internal node annotations are as per Fig. 3. Scale bar units are number of substitutions per site.

The third East/Southeast Asian sub-lineage was L4.5, with 270 genomes forming a sub-clade identified by TreeBreaker (Fig. 5) (two basally branching genomes within this clade were assigned as L4 by tb-profiler). There were 101 Thai, 99 Chinese, 46 Vietnamese, 13 Indonesian, six other Asian and five European genomes in this sub-clade. The MRCA of this sub-clade was assigned to China (89%) in AD 945 (90 % CI 873–972), while the parent of the MRCA was assigned to Europe (92%). According to the SIMMAP analysis, there were two high-confidence migrations from China into Thailand, which resulted in two Thai sub-clades of 26 and 13 genomes respectively. The MRCAs for these two Thai clades were AD 1704 and AD 1679 respectively. There were 10 other China to Thailand migrations which resulted in smaller sub-clades of two to eight sampled genomes, containing a total of 34 Thai samples. In contrast, 37 of the 46 (80 %) L4.5 sampled in Vietnam descend from a single introduction from China with an MRCA of AD 1446. In the sub-sampling analysis, 85.6 % of replicates placed the MRCA in China, and 14.4 % in the UK.

## Global sub-clades

### L4.1.2 and L4.1.2.1

Twenty five per cent of Lineage 4.1.2 and its sub-lineage L4.1.2.1 were from Southeast Asia (Table S1: a full line list of all L4 read-sets included in this analysis; Table S2, Fig. 2). The MRCA of a sub-clade of 284 genomes identified by TreeBreaker was placed in Vietnam (64%) or Europe (21%) by SIMMAP and dated to AD 1078 (90% CI 1028–1093), while the parent of the MRCA was placed in Europe (74%) or Vietnam (16%) (Fig. S1). SIMMAP confidently placed the MRCA (AD 1475, 90% CI 1430–1475) of a sub-clade of 255 of the 284 genomes in Vietnam (96% confidence), but then a sub-clade of 234 of these 255 genomes had an MRCA placement in Europe (89% confidence) in AD 1537 (90% CI AD 1505–1538). Of the 64 Indonesian L4.1.2.1 genomes, 37 were in a sub-clade of 64 genomes for which the MRCA was probably in Indonesia (70%) in AD 1653 (90% CI 1622–1658) while the parent node of the MRCA was probably in Europe (71%). There were three sub-clades that were high-confidence (>80%) migrations from Europe to Vietnam, leading to a total of 17 sampled Vietnamese genomes.

### L4.4.1.1

L4.4.1.1 is a global lineage, with 26 % of genomes coming from Southeast Asia. There were two Southeast Asian clades with more than two isolates within L4.4.1.1; one sub-clade consisted of five Vietnamese isolates. SIMMAP identified a South American location for the parent node of this Vietnamese sub-clade, as it was nested within a sub-clade of Brazilian TB genomes (Fig. S2). The second Southeast Asian L4.4.1.1 sub-clade consisted of eight Indonesian isolates, for which the parent node of the MRCA was placed in North America by SIMMAP and dated to AD 1702 (90 % CI 1671–1723), as many of its close phylogenetic neighbours were isolated in Canada.

### L4.4.1.2

L4.4.1.2 is a small clade that was 65 % Southeast Asian, with 54 % from Indonesia and 11 % from Vietnam. While this lineage is well established in Indonesia, there was considerable uncertainty as to the location of the parent node of the MRCA, with SIMMAP analysis identifying South America (48%) or Europe (38%) as being the most plausible locations of origin.

### L4.8

L4.8 was 28 % Southeast Asian, with Indonesia contributing 12%, Vietnam 11 % and Thailand 4 %. There was one sub-clade of 47 genomes for which SIMMAP identified the most likely location of the MRCA as Indonesia (76%), but the location of the parent of the MRCA was uncertain (Europe 42 % or Indonesia 36%). There was another sub-clade with seven Indonesian genomes which represented a high-confidence (99%) migration from Europe to Indonesia. One Vietnamese sub-clade of seven genomes was the result of a migration from Europe (99%), while another sub-clade of four genomes was from Indonesia (94%). There was a sub-clade of five Thai genomes which was also a direct migration from Europe (100%).

### L4.3

There are only 35 L4.3 genomes in our analysis, but 74 % of them were Indonesian. Therefore, although it did not meet our definition of a Southeast Asian sub-clade it was still dominated by Southeast Asian genomes. While the MRCA of a sub-clade of 25 of them was confidently placed in Indonesia (97 % confidence), the parent of the MRCA was either in South America (60%) or in Europe (27%).

### L4.3.4.1 and L4.3.4.2

L4.3.4.1 and L4.3.4.2 are associated with South America, with 56 and 57 % of their genomes coming from that continent (Fig. S3). Two sub-clades of Southeast Asian genomes were both from the Philippines; one of 11 Filipino genomes with an MRCA date of AD 1658 where the parent of the MRCA was placed in South America (99%), and a sub-clade of 17 genomes, of which eight were Filipino, and where the MRCA was in the Philippines (100%) in AD 1806 and the parent of the MRCA was either in South America (70%) or in the Philippines (30%).

## Discussion

Our findings extend the idea of the ‘out of Europe’ spread of *M. tuberculosis* L4 by showing that there were historical movements of *M. tuberculosis* L4 between countries in East and Southeast Asia. The sub-clades of *M. tuberculosis* L4 transmitted between East and Southeast Asian countries continue to be important contributors to the burden of *M. tuberculosis* L4 disease in the region. While the limitations of the current sampling frame cause uncertainty around the precise order in which L4 migrated between countries, the data suggest that China was the intermediary between Europe and Southeast Asia for two of the Asian sub-clades, L4.2.2 and L4.5 (Fig. S8). It also appears that for L4.2.2 and L4.5, Thailand was a ‘sink’, receiving importations from both China and Vietnam (Fig. S8). For L4.4.2, there is a possibility that Thailand or Vietnam received the original importation from Europe, and subsequently exported to China and the other country.

A major strength of our study is that we have carried out a combined analysis of *M. tuberculosis* L4 datasets from every Southeast and East Asian country for which they are available and placed them into a global context. Furthermore, we have employed a novel approach of using TreeBreaker as a screening tool to identify clades of interest for more in-depth phylogeographical investigation in this large dataset. We grouped these identified clades into three ‘kinds’ that represented different patterns of presence in Southeast Asia. Three sub-lineages were identified (L4.2.2, L4.4.2 and L4.5) in which most of the genomes belonging to that sub-lineage were from East/Southeast Asia. These sub-lineages were probably introduced into East/Southeast Asia 500–1000 years ago [9, 15], and are potentially undergoing ‘niche’ adaptation to their host population [67]. The second kind of sub-clade was Southeast Asian sub-clades within global lineages. These probably represent introductions within the last 500 years of ‘generalist’ sub-lineages into the region. Finally, we identified ‘reversions’ – these were so termed because L4 is thought of as a European lineage, and these sub-clades represent exportations of a ‘European’ lineage from Asia back to Europe, i.e. they have ‘reverted’ back to Europe. The main limitation of our analysis is that phylogeographical investigations are very susceptible to sampling bias so we should be cautious in our interpretation. This is especially true when analysing data that come from studies with very different sampling frames, such as those analysed here. In the sub-sampling analysis, 14 % of sub-samples placed the MRCA of L4.5 in the UK, reflecting the sensitivity of phylogeographical analysis to the small number of relatively deeply branching L4.5 genomes from the UK. The uncertainty in the location of the MRCA of L4.4.2 in the sub-sampling analysis was mirrored by the uncertainty in the SIMMAP assignment for this MRCA and suggests a complex migratory history that cannot be explained by the currently sampled genomes. Another limitation is that few countries in our analysis have nationally representative genome collections, with most represented by genomes from only one city or region. The dating analysis was limited by the lack of availability of the year of isolation of many genomes in our analysis, leaving us to rely on published substitution rates for L4 [44].

Our study extends the findings of recent studies. Brynildsrud *et al*. and Holt *et al*. found that L4 has been introduced to Vietnam multiple times, with the first time being from Europe at the beginning of the 13^th^ century [9, 25]. However, these analyses did not include genomes from Thailand, China or Indonesia, and so their ability to identify intra-Asian migrations was limited. Liu *et al*. analysed Chinese and Vietnamese L4, but their focus was on L4 within China, and they only noted that ‘closest branches to the strains sampled from Vietnam were mostly collected in South China’ [15]. The dates we identified for the arrival of L4.2.2, L4.4.2 and L4.5 in East/Southeast Asia were within the 95 % CIs of previous estimates [15]. An analysis of all lineages of *M. tuberculosis* in Africa and Eurasia found that Southeast Asia was the most connected region in terms of *M. tuberculosis* migrations globally [10]. While O’Neill *et al*. found this to be primarily driven by Lineage 2, the high level of connectedness in Southeast Asia is also reflected in the dynamic picture of L4 migration we have identified. The impact of historical population movements on the distribution of L4 has been well described [8, 9, 47]. From our analysis, we can see the impact of pre-colonial, colonial and post-colonial relationships in the L4 phylogeny, with ‘migrations’ from South America to the Philippines (L4.3.4.1 and L4.3.4.2), and between Indonesia and the Netherlands (L4.8). The South America to the Philippines migrations could have been true direct migrations, mediated by colonial trade links such as the Manilla galleons which sailed between the Philippines and Spanish colonies in central and South America, or could represent migration from a common, unsampled source (i.e. historical Spain). This explanation is consistent with our dating analysis, which identified that the MRCAs of the Filipino sub-clades were in AD 1658 and AD 1806, during the period of Spanish colonization. The evidence of migrations from Indonesia to the Netherlands is consistent with the historical Dutch colonization of Indonesia, and demographic and cultural connections that persist to this day. L4.4.1.1 has been reported as transmitted from French-Canadian fur traders to Western Canadian First Nations people in the 18^th^ and 19^th^ centuries [68], and in Polynesia, linked to European whalers and other merchants [69]. Here, we report that this lineage is a major cause of *M. tuberculosis* L4 cases in Indonesia. This adds to the remarkably diverse destinations of this well-travelled sub-lineage. While the SIMMAP analysis identified a North America to Indonesia transfer, based on the Canadian genomes sampled by Pepperell *et al*., a more historically congruent explanation could be speculated as both the Canadian and Indonesian sub-clades originating from a clonal population in France, with the possibility that the transfer to Indonesia could be connected to the French administration of Indonesia between AD 1806 and 1811 [70]. This is consistent with our dating analysis, which placed the MRCA of the Indonesian sub-clade of L4.4.1.1 in AD 1805. Our dating analysis showed that the MRCA of the Indonesian and French-Canadian sub-clade was in AD 1702, which was within the 95 % highest probability density (HPD) of previous estimates for this sub-clade [69]. As the French *M. tuberculosis* population underwent a major bottleneck in the 20^th^ century, it is unlikely that we will have strong phylogenetic evidence of seeding from France without historical French genomes.

One major implication of our findings, building on those of Liu *et al*. and Brynildsrud *et al*., is that multiple sub-lineages of L4 have been circulating in Asian populations for hundreds of years. Considering the hypothesis that *M. tuberculosis* is undergoing host population-specific adaptation [67, 71], in future work it would be interesting to look for signals of adaptation to that specific host population.

While the findings reported here enrich our understanding of L4 in Asia, having consistent sampling frames between different countries would increase the certainty of the conclusions we can draw. Therefore, in future research, carrying out structured surveys such as those of Liu *et al*., or using unique isolate collections from those such as National TB Prevalence surveys, would provide a more comprehensive picture of TB in the region. In addition, the analysis of historical TB genomes has improved our understanding of the 19^th^ century European TB epidemic. Having further contextual genomes from a broader swathe of 19^th^ century Europe would provide very interesting context for these samples.

## Supplementary Data

Supplementary material 1

Supplementary material 2
