# Supplementary material for: Distribution and origins of Mycobacterium tuberculosis L4 in Southeast Asia
Source: Microb Genom. 2023 Feb 2;9(2):mgen000955. doi: 10.1099/mgen.0.000955 (PMC9997747; doi:10.1099/mgen.0.000955)
Supplement: Supplementary material 1 [file mgen-9-955-s001.pdf]

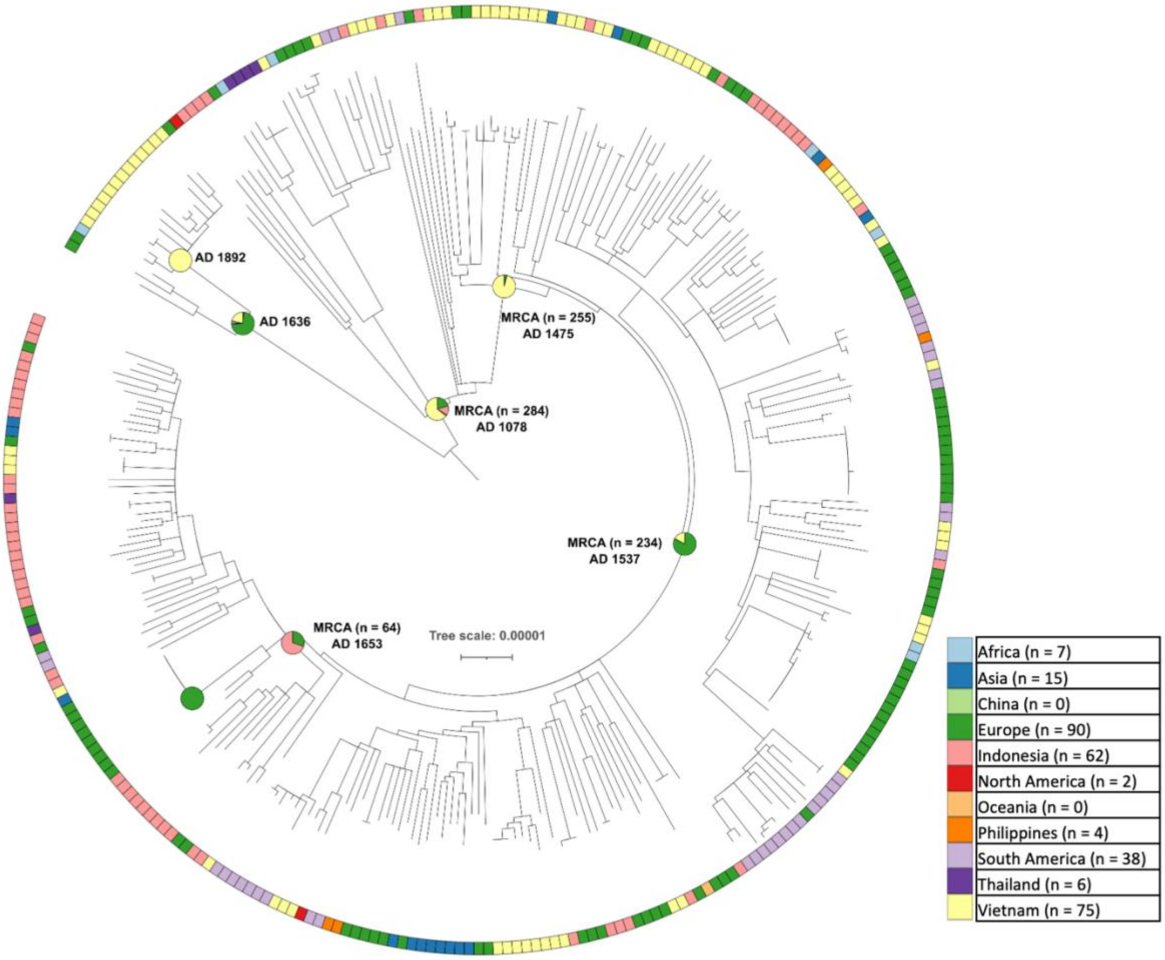

Supplementary Figure 1: A maximum likelihood phylogeny of lineage 4.1.2 and descendants. Selected nodes are annotated with pie charts reflecting the placement of that node in SIMMAP analysis.

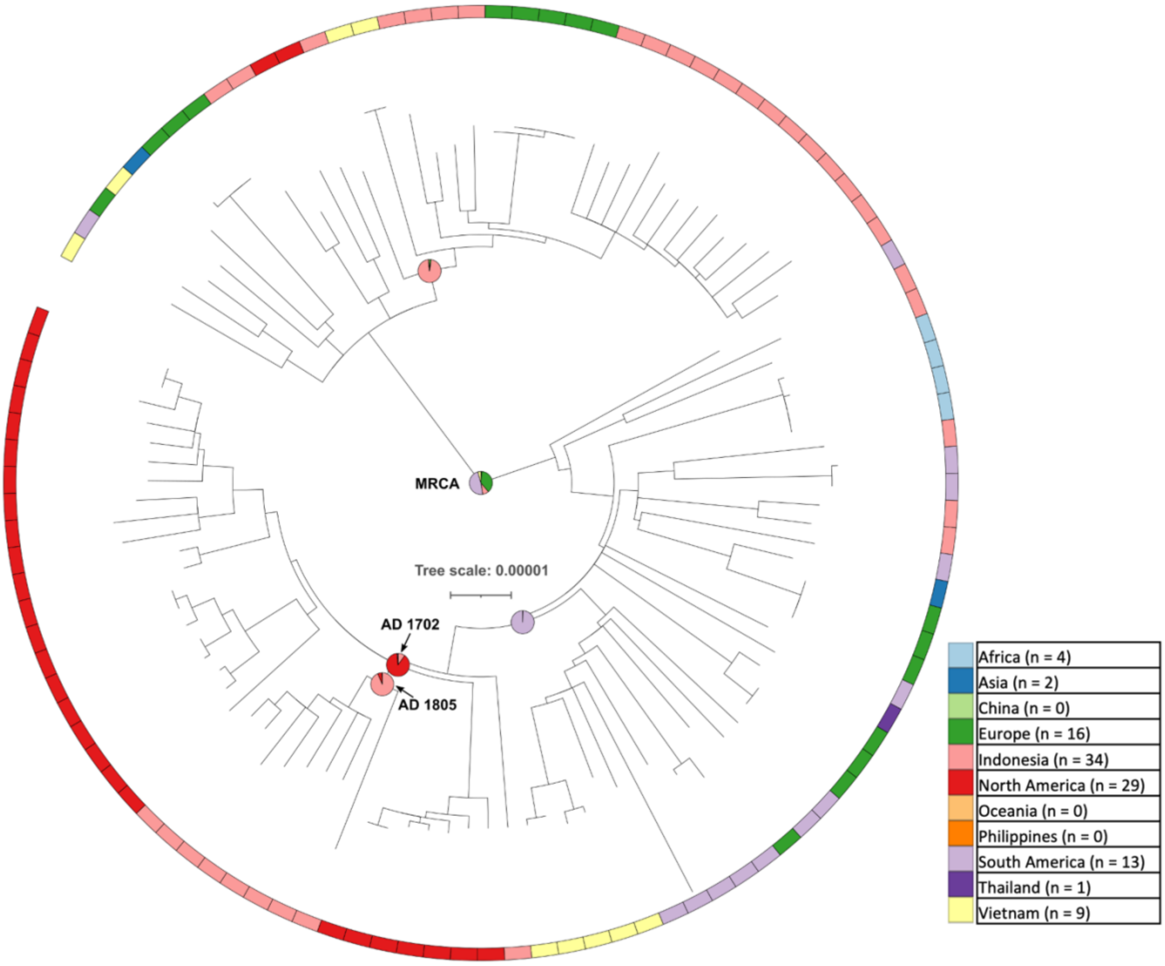

Supplementary Figure 2: A maximum likelihood phylogeny of lineage 4.4.1.1 and descendants. Selected nodes are annotated with pie charts reflecting the placement of that node in SIMMAP analysis.

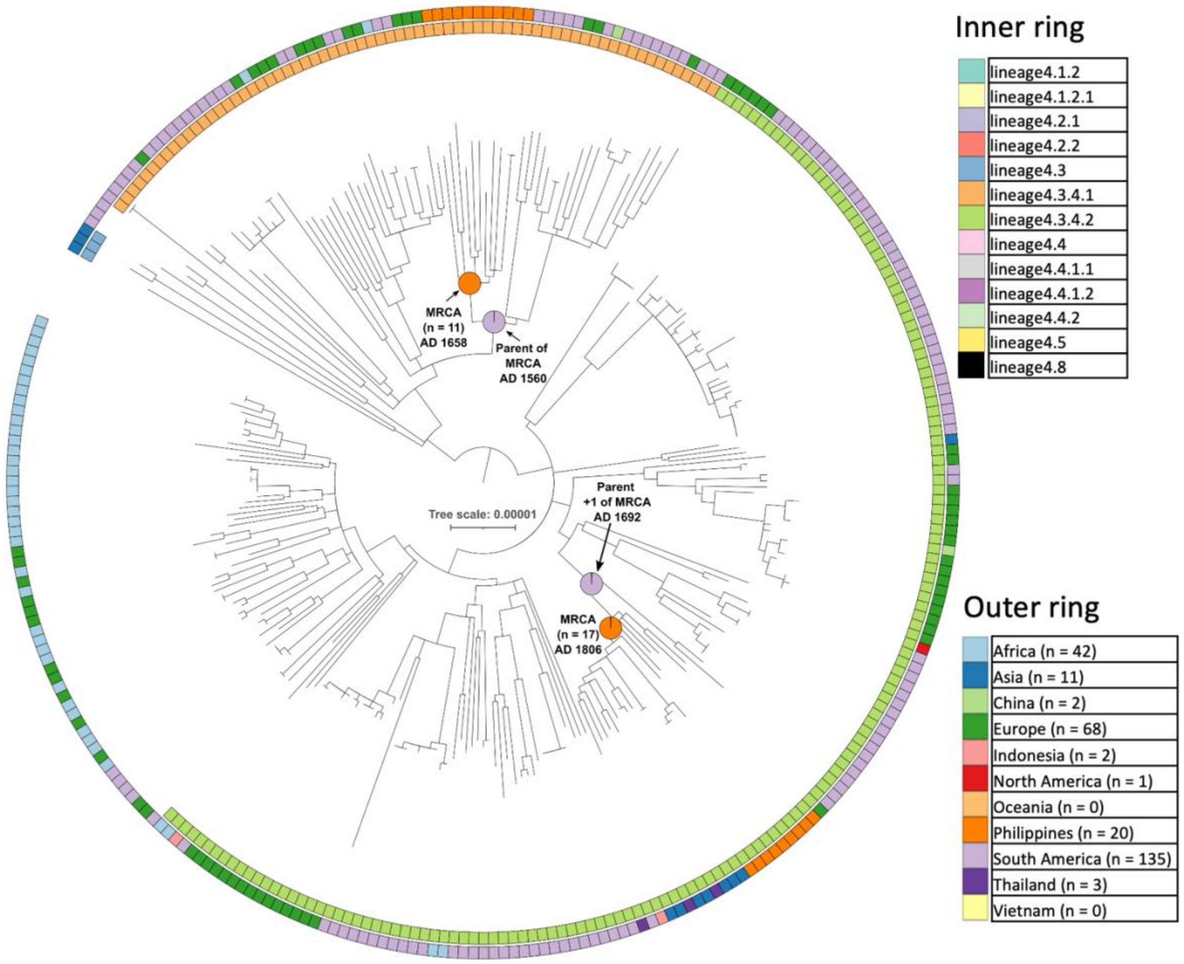

Supplementary Figure 3: A maximum likelihood phylogeny of lineage 4.3.4 and descendants. Selected nodes are annotated with pie charts reflecting the placement of that node in SIMMAP analysis

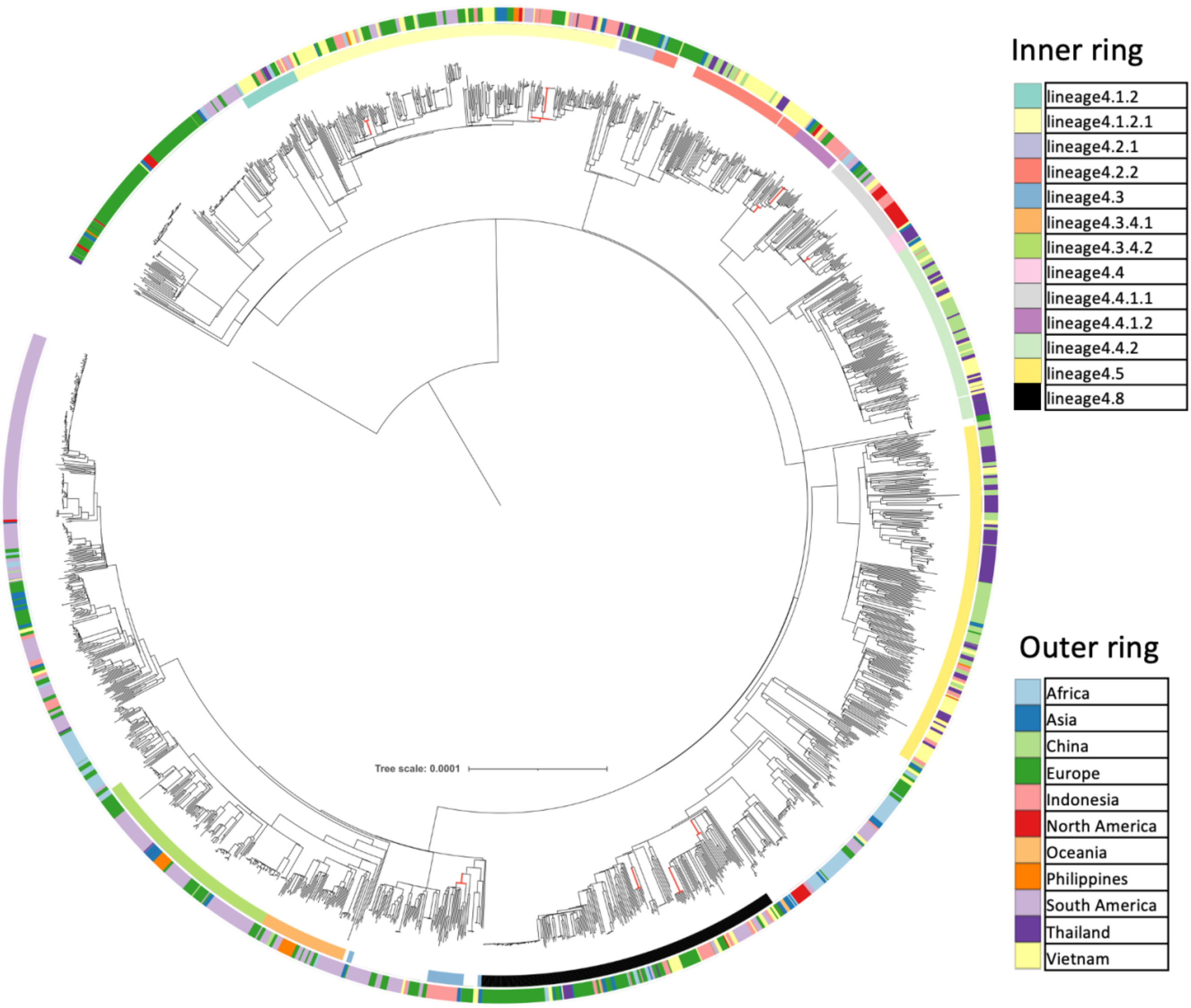

Supplementary Figure 4: Results of TreeBreaker analysis for Indonesia. A maximum likelihood phylogenetic tree of 2257 M. tuberculosis L4 genomes. TreeBreaker identifies branches of the tree where the distribution of tips with a specific phenotype differs from the overall distribution (i.e. where there are a greater or fewer number of genomes with that phenotype than the expected number). Branches are coloured in red where TreeBreaker identified that there was a posterior probability greater than 0.5 that a change in the distribution of tips from Indonesia occurred on that branch. The outer ring represents the country of origin of each tip.

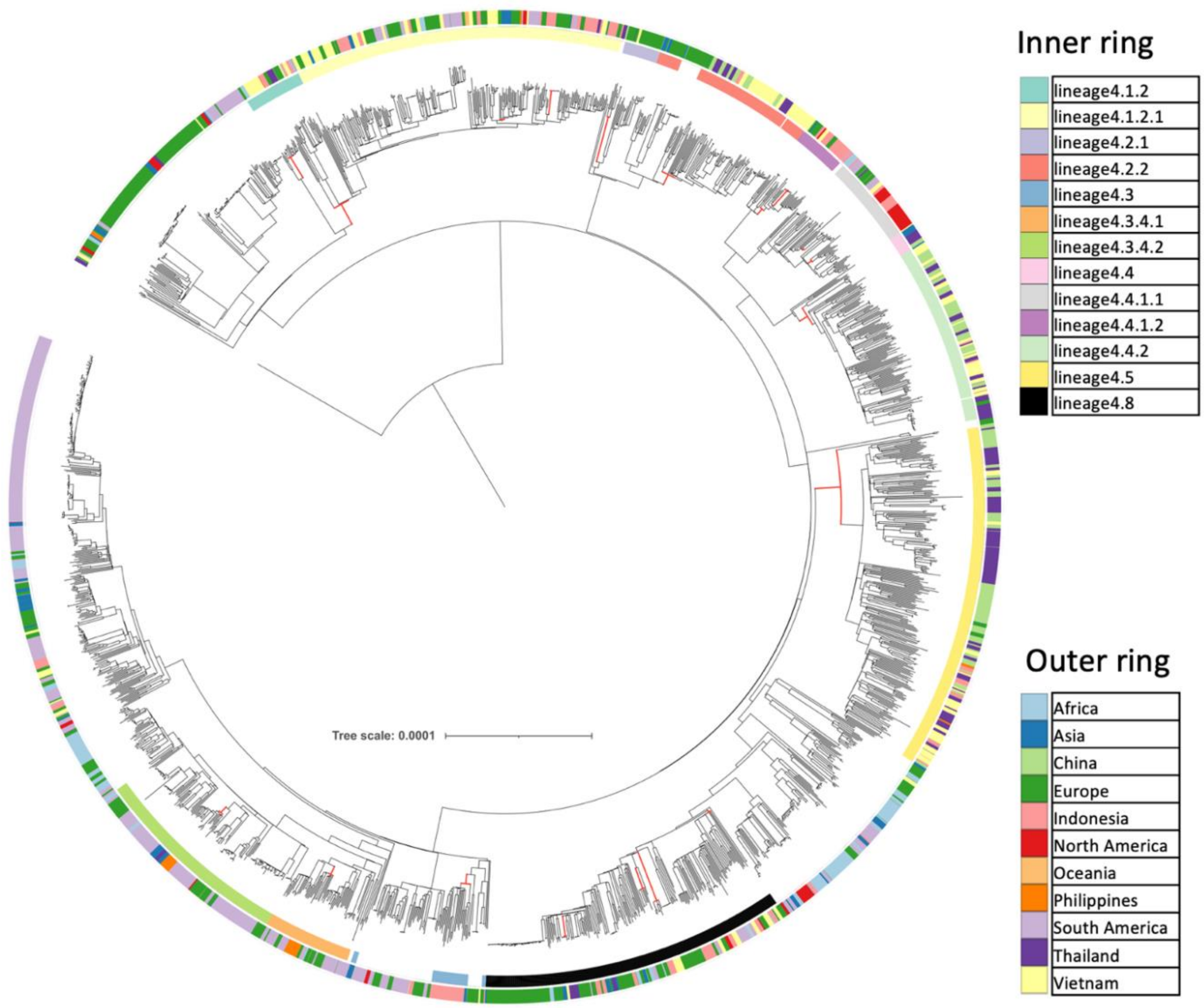

Supplementary Figure 5: Results of TreeBreaker analysis for Thailand. A maximum likelihood phylogenetic tree of 2257 M. tuberculosis L4 genomes. TreeBreaker identifies branches of the tree where the distribution of tips with a specific phenotype differs from the overall distribution (i.e. where there are a greater or fewer number of genomes with that phenotype than the expected number). Branches are coloured in red where TreeBreaker identified that there was a posterior probability greater than 0.5 that a change in the distribution of tips from Thailand occurred on that branch. The outer ring represents the country of origin of each tip.

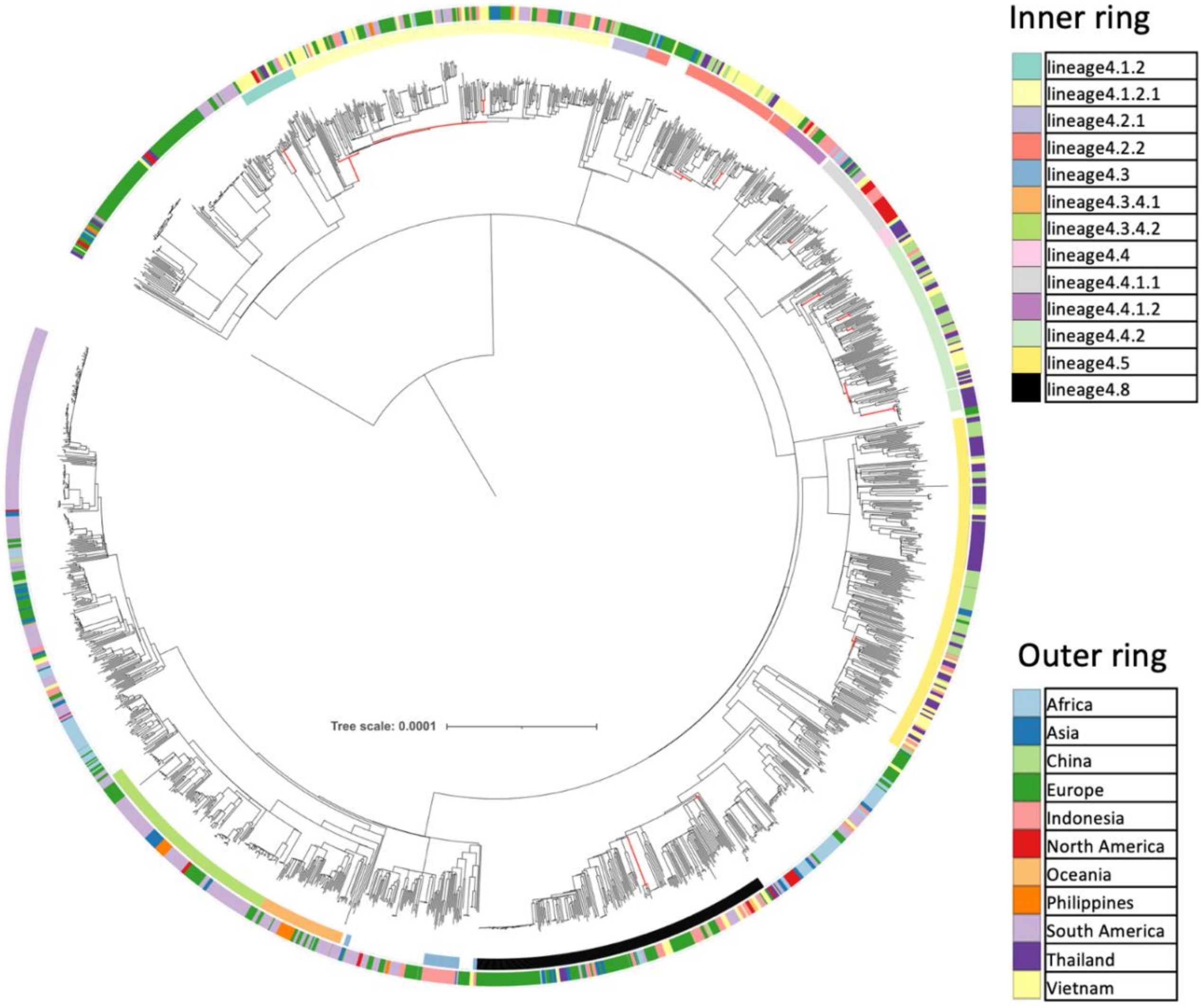

Supplementary Figure 6: Results of TreeBreaker analysis for Vietnam. A maximum likelihood phylogenetic tree of 2257 M. tuberculosis L4 genomes. TreeBreaker identifies branches of the tree where the distribution of tips with a specific phenotype differs from the overall distribution (i.e. where there are a greater or fewer number of genomes with that phenotype than the expected number). Branches are coloured in red where TreeBreaker identified that there was a posterior probability greater than 0.5 that a change in the distribution of tips from Vietnam occurred on that branch. The outer ring represents the country of origin of each tip.

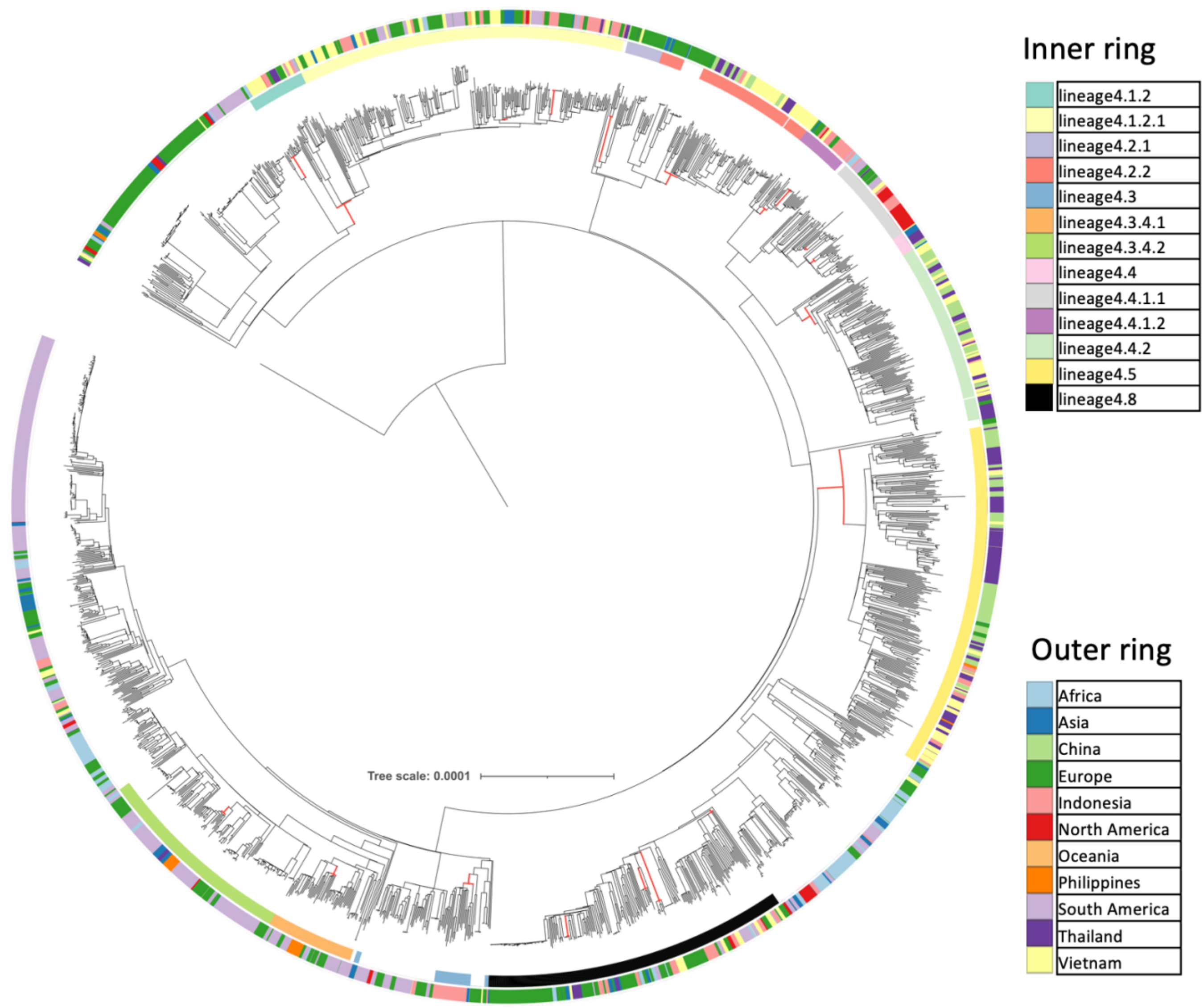

Supplementary Figure 7: Results of TreeBreaker analysis for a combination of China, Indonesia, Thailand and Vietnam. A maximum likelihood phylogenetic tree of 2257 M. tuberculosis L4 genomes. TreeBreaker identifies branches of the tree where the distribution of tips with a specific phenotype differs from the overall distribution (i.e. where there are a greater or fewer number of genomes with that phenotype than the expected number). Branches are coloured in red where TreeBreaker identified that there was a posterior probability greater than 0.5 that a change in the distribution of tips from China, Indonesia, Thailand and Vietnam occurred on that branch. The outer ring represents the country of origin of each tip.

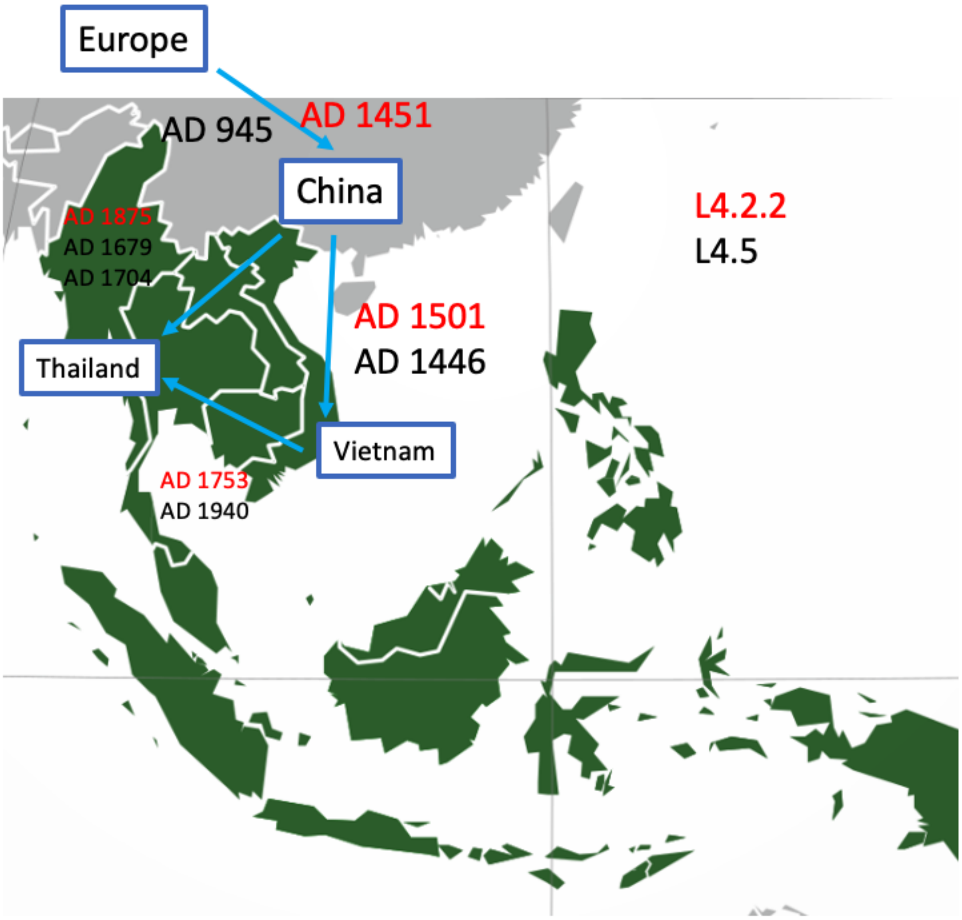

Supplementary Figure 8: A schematic diagram of the major routes of migration observed in M. tuberculosis phylogenies for sub-lineages L4.2.2 and L4.5. The migration routes of L4.4.2 are too uncertain to present in this fashion. The dates are the date of arrival in each country, dates in red are for L4.2.2, dates in black are for L4.5. Adapted from https://commons.wikimedia.org/wiki/File:Southeast_Asia_(orthographic_projection).svg.
